# Report on Insanity

**Published:** 1867-06

**Authors:** 


REPORT ON INSANITY.

Dr. C. A. Walker, of Boston, read the report of Dr. J. Ray, of Providence, R. I., on insanity. It was an able and interesting paper.

Dr. Chipley, of Lexington, Ky., offered some remarks in vindication of the Superintendents of Insane Asylums, with reference to their connection with this association. He referred to the fact that there were five such Superintendents present, who were a larger proportion of their class than the representatives of any other class of the medical profession present.

The report of Dr. Ray was referred to the Committee of Publication.

